# A Review of New High-Throughput Methods Designed for Fluorescence Lifetime Sensing From Cells and Tissues

**DOI:** 10.3389/fphy.2021.648553

**Published:** 2021-04-26

**Authors:** Aric Bitton, Jesus Sambrano, Samantha Valentino, Jessica P. Houston

**Affiliations:** Department of Chemical and Materials Engineering, New Mexico State University, Las Cruces, NM, United States

**Keywords:** fluorescence lifetime, high-throughput approaches, fluorescence lifetime imaging microscopy, flow cytometry, frequency domain, time domain

## Abstract

Though much of the interest in fluorescence in the past has been on measuring spectral qualities such as wavelength and intensity, there are two other highly useful intrinsic properties of fluorescence: lifetime (or decay) and anisotropy (or polarization). Each has its own set of unique advantages, limitations, and challenges in detection when it comes to use in biological studies. This review will focus on the property of fluorescence lifetime, providing a brief background on instrumentation and theory, and examine the recent advancements and applications of measuring lifetime in the fields of high-throughput fluorescence lifetime imaging microscopy (HT-FLIM) and time-resolved flow cytometry (TRFC). In addition, the crossover of these two methods and their outlooks will be discussed.

## INTRODUCTION

The fluorescence lifetime is a unique optical parameter that has been exploited for many decades for a variety of biological applications. The fluorescence lifetime is defined as the average time a molecule spends in an excited state prior to returning back to its relaxed ground state. When measured, most organic fluorophores have a fluorescence lifetime that ranges between 100 ps and 15 ns [1]. When this lifetime is measurable, the information that it encodes reveals direct or indirect changes of the fluorophore itself, as well as its surroundings. Every fluorescence molecule has a unique fluorescence lifetime, and the value of the lifetime is reflected by the chemical and physical characteristics of the microenvironment in which the fluorophore resides [2]. Moreover, the fluorescence lifetime is independent of fluorescence brightness; thus, the fluorescence lifetime can be detected from dim fluorescence signals, making accurate measurements of fluorophores that are dim or in low concentrations possible. With these unique characteristics, the lifetime of a fluorophore has become quite valuable in life sciences. For example, Förster resonance energy transfer (FRET), a phenomenon where fluorescence properties are altered when two fluorophores are in close proximity (<10 nm), can be more accurately quantified using lifetime compared to other fluorescence properties [3]. This and other advantages have led to a variety of novel bio-inspired applications where the resulting lifetime data reveal new discoveries. Since the fluorescence lifetime continues to be a sought-after optical parameter, we use this review to focus on technologies that measure fluorescence lifetime with a focus on higher-throughput systems.

The applications of fluorescence lifetime measurements range significantly. Some examples include separating fluorophores that have similar spectra but different lifetimes [4–13], exploiting the difference in lifetime of free and bound metabolites for metabolic study and diagnosis [14–24], using FRET to screen for binding or inhibition of exogenous and endogenous molecules of interest [2, 5, 25–35], evaluating the conformation and stability of proteins or other molecules in varying environments [31, 36], or even using lifetime as an additional parameter in fluorescent inks for anti-counterfeiting [37], though this list is far from exhaustive. New methods and technologies continue to be discovered, further establishing the wide-reaching significance and potential of fluorescence lifetime. Along with these distinct advantages and potential, there are unique challenges present in measuring fluorescence lifetime. Most of these difficulties arise from the short timescale of fluorescence decay, though these are now much easier to overcome given the current state of the modern laser, microchip, and other optoelectronic technologies. Ranging from hundreds of picoseconds to tens of nanoseconds, recording the process requires high-speed excitation, detection, and data acquisition hardware and techniques.

**FIGURE 1 |:**
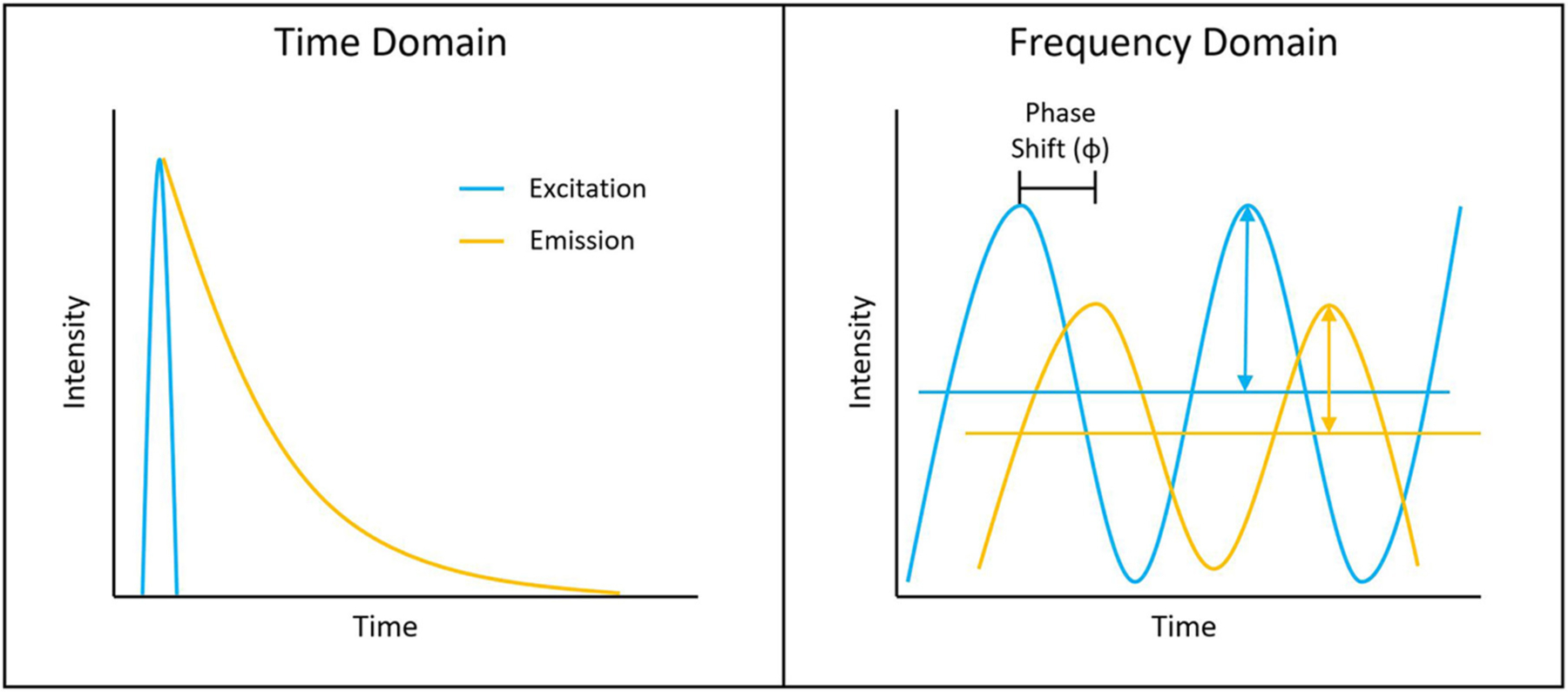
Depiction of typical time-domain data **(left)** and frequency-domain data **(right)**. Lifetime determination in the time domain is a function of the decay curve created by a histogram of photon arrival times. For the frequency domain, the lifetime is a function of the phase shift between the laser excitation and fluorescence emission light.

**FIGURE 2 |:**
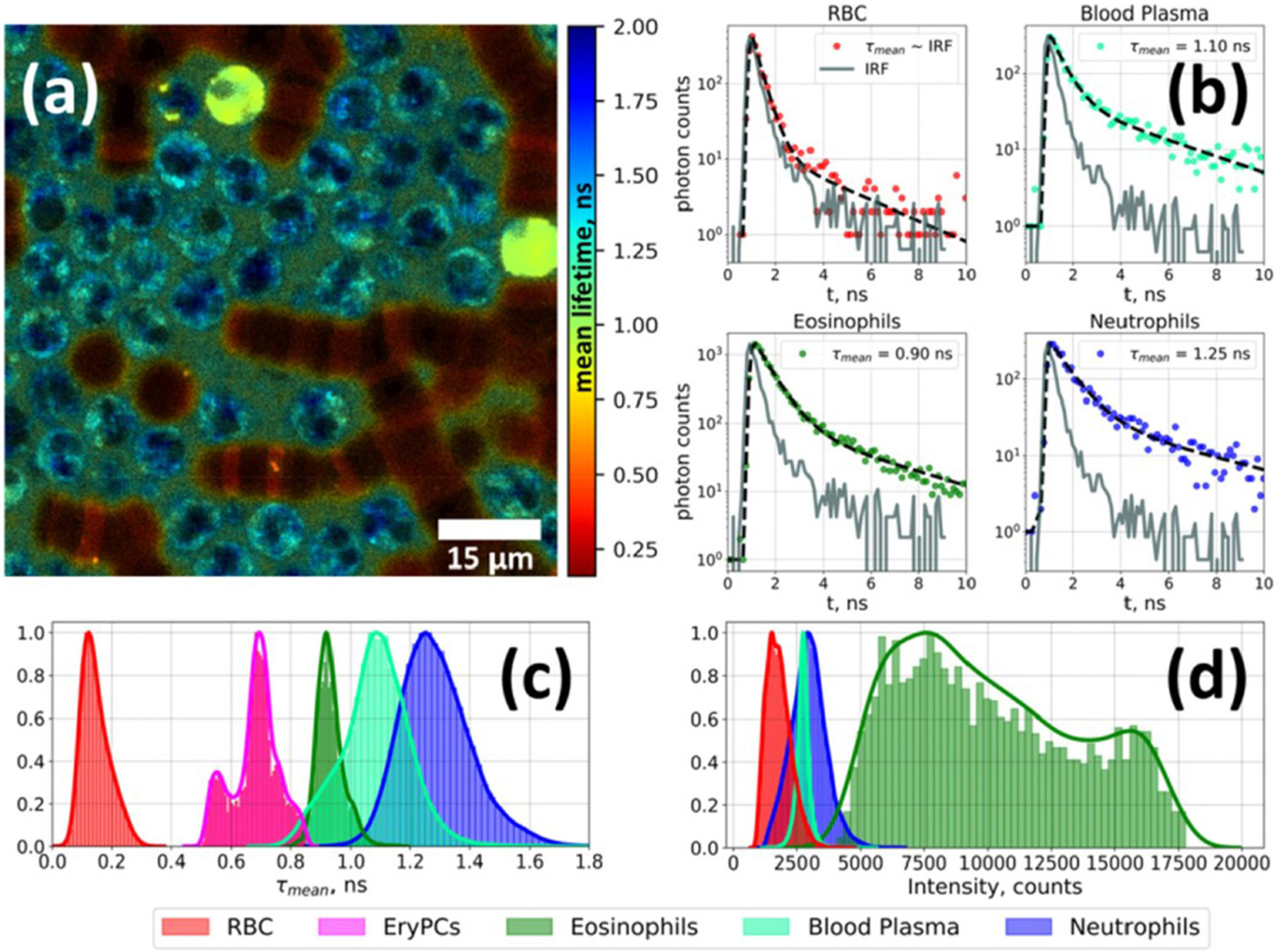
Fluorescence lifetime imaging microscopy image of autofluorescence from a white blood cell-enriched sample excited at 402 nm and collected at >520 nm **(a)**. Fluorescence decay curves for each blood-cell type was generated using time-correlated single-photon counting (TCSPC) along with a biexponential decay curve fit **(b)**. Plots of average fluorescence lifetime **(c)** and integral fluorescence intensity for each cell type **(d)**. Adapted with permission from [23] © The Optical Society.

Measuring the fluorescence lifetime is traditionally approached in one of the two ways: with optoelectronics that involves either time-domain or frequency-domain measurements (see Figure 1). In the time domain, the timing of fluorescence photons is directly measured, often against a pulse of excitation light, as if the excitation light is starting a clock or a timer. A few of the most common time-domain methods are time-correlated single-photon counting (TCSPC), time gating, and direct waveform recording or pulse sampling. TCSPC uses fast electronics and/or fast detectors to measure the arrival time of individual photons. These arrival times of the photons are then gathered into time bins and visualized with a histogram. As shown in Figure 2, less photons will be present in the time bins as the fluorescence decays, causing the shape of the histogram to be representative of the decay curve and showing graphically the probability density function of the photon arrival times [38]. Though TCSPC can be very accurate and have a high temporal resolution, the throughput is heavily dependent on the ability of the detector and electronics to avoid photon pile-up. This is caused by the dead time, a period after initial triggering where no photons will be recorded until the detector and electronics are re-primed. Reducing dead time has been a main source of innovation for increasing throughput and will be discussed further along with detection devices. Unlike TCSPC, time gating does not record the fluorescence decay in terms of individual photon timing but handles the measurements in short sections of time that cover a set portion of the decay curve, stitching those sections together digitally for the full picture. Direct waveform recording uses high-speed digitizers and records the entire waveform of the fluorescence signal, from which the lifetime information is then extracted. The latter two methods are not as affected by, or avoid entirely, the issue of detector dead time and thus can have faster acquisition speeds, but they are generally not as sensitive or accurate as TCSPC.

To measure fluorescence lifetime in the frequency domain, the excitation source intensity is modulated, causing the resulting fluorescence signal to follow the same pattern. However, due to the fluorescence decay time, the fluorescence signal will be shifted compared to the excitation source signal. This phase shift is directly proportional to the fluorescence lifetime and can be extracted mathematically, i.e., using Fourier transforms [39]. Since this method employs continuous fluorescence detection rather than pulsed or gated detection seen in most time-domain methods, photon pile-up does not occur and frequency-domain lifetime detection can be used at much higher throughputs, such as those seen in flow cytometry [40]. However, the performance is more dependent on the brightness of the sample and measurements are generally lower in accuracy, resolution, or both compared to what is achievable in the time domain.

Both time-domain and frequency-domain high-throughput methods will be discussed in the context of fluorescence lifetime imaging microscopy (FLIM) and time-resolved flow cytometry (TRFC) in the following sections of the review. A summary of the lifetime techniques to be discussed is provided in Table 1.

## HIGH-THROUGHPUT FLIM (HT-FLIM)

**FIGURE 3 |:**
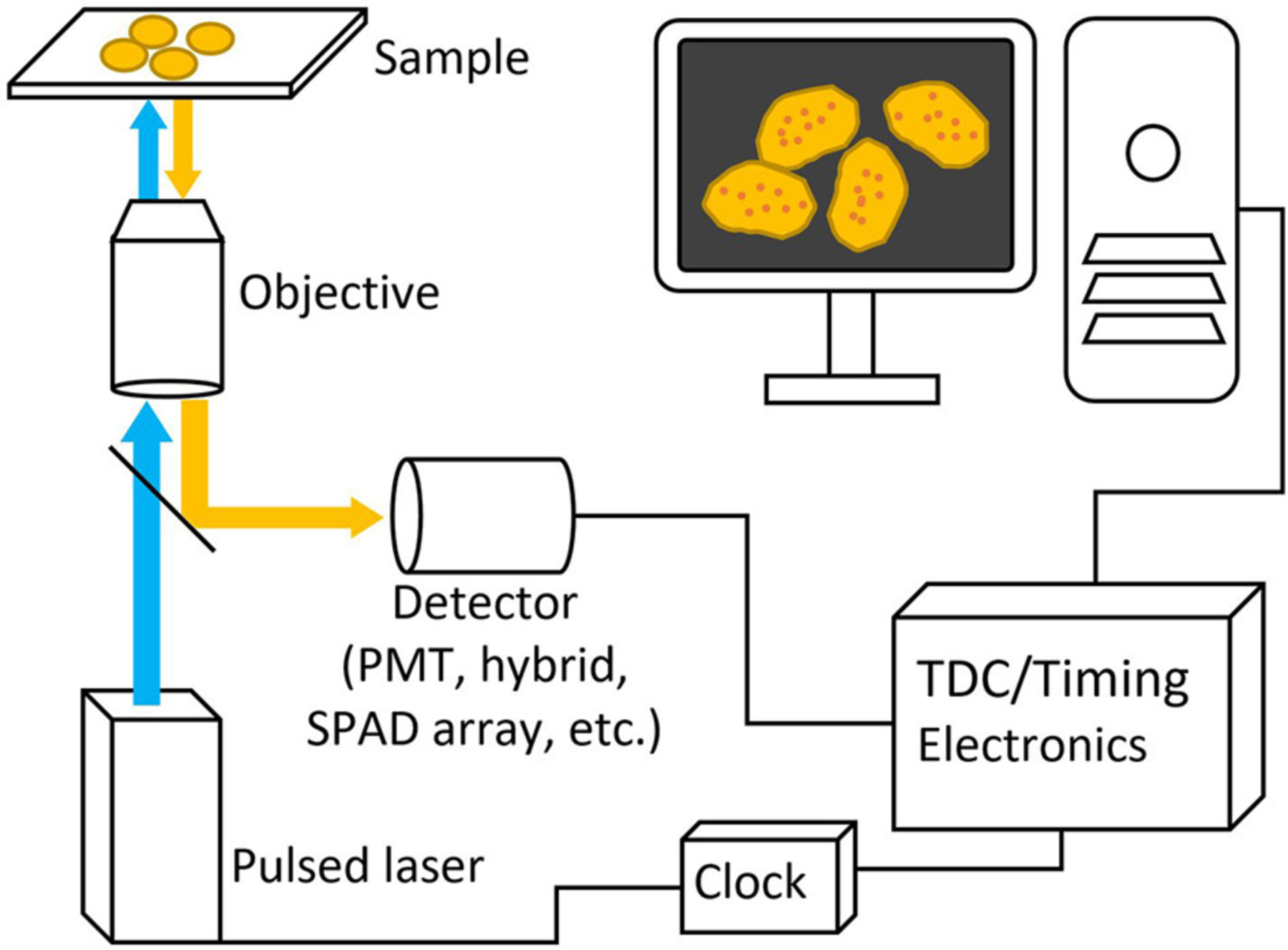
Basic schematic picture of a generalized FLIM system. Pulsed excitation light passes through the microscope to the sample and the excitation light travels back through and is deflected to a detector with a dichroic mirror. Both laser and detector setups are then connected to timing electronics for lifetime calculation.

The large field of FLIM that encompasses many different methods, instruments, and applications is attributed to the long-standing history of fluorescence microscopy. Increasing the speed and throughput capabilities of FLIM systems reduces the imaging time and can increase multiplexing capabilities, magnifying its usefulness in many applications, particularly those that are time-sensitive, such as real-time imaging or clinical diagnosis. However, increasing speed and/or throughput usually entails compromises, typically in the form of reduced temporal or spatial resolution and accuracy. The opposite is also generally true; if high accuracy and resolution are desired, then image acquisition time is long. These compromises can often be a function of the microscopy platform used, such as widefield vs. confocal, or be a result of the detection and data acquisition systems. Figure 3 contains a schematic picture of a general FLIM setup. This review focuses on more recent systems from literature in the past decade or on those leading the advancement in HT-FLIM methods and technology with discussions on capabilities, innovations, trade-offs, and current applications. Since TCSPC is to some extent a gold standard and is heavily utilized in FLIM [11], discussion of systems and applications will be separated by those that are TCSPC-based and those that are not, including frequency-domain FLIM.

### TCSPC in HT-FLIM

TCSPC is a long-used and extensively researched method in FLIM due to its hardware availability, high sensitivity, resolution, and various possible implementations. The main setback for TCSPC with regard to throughput is photon pile-up caused by dead time in the detector and timing electronics, during which valuable information is lost from the photons that are not detected. As a result, much of the research involved in increasing TCSPC throughput is centered on either decreasing the dead time or finding ways to mitigate the effects of dead time and reduce photon pile-up. Traditionally, photomultiplier tubes (PMTs) are used, operating in single-photon counting mode with separate electronics for signal processing and analysis. In the context of TCSPC, this only provides one channel for photon timing with limited throughput [41]. One solution to this issue is parallelization, which theoretically can be applied to any detector/timing electronic setup, given there is enough funding, space, and power available. In 2012, however, one group presented a more elegant, highly parallelized solution capable of 10× the photon counting rates of traditional TCSPC using an iteration of their novel single-photon avalanche diode (SPAD) array that included an accompanying array of time-to-digital converters (TDCs) and circuitry to perform lifetime estimations in real time on one chip made using a CMOS process [42]. Since then, the technology has been continuously improved through increasing the SPAD array pixel count [43], creating a multi-focal system to increase resolution and detector efficiency, and moving from a widefield to a laser-scanning approach for live FRET studies [26]. The attractiveness of having a scalable, accurate, all-in-one TCSPC solution built into a microchip has encouraged others to develop their own SPAD array systems using similar CMOS processes for specific applications, such as a high-speed, large pixel count camera for fluorescence lifetime imaging at high frame rates [44]. For a more in-depth look at the history, architecture, and application of SPADs in FLIM and other fields, the recent review by Bruschini et al. is recommended [45].

Although integrated silicon photomultiplier SPAD arrays are highly versatile and provide great temporal resolution, they are not a universal answer for TCSPC. Apart from the standard PMT and counting hardware setup, there are hybrid detectors (called hybrid PMTs) that combine the photocathode and vacuum tube from PMTs with avalanche diodes, similar to those seen in SPADs but operated at lower voltages, resulting in much lower detector dead times (<1 ns) compared to PMTs and SPADs alone (>10 ns) [41]. When paired with fast timing electronics, such as fast TDCs, systems employing hybrid detectors for TCSPC can have count rates comparable to some SPAD arrays while being simpler and made entirely from off-the-shelf products [11]. As a result, system complexity and cost can be more dependent on the timing electronics than the hybrid PMTs and be chosen to fit the needs of the application.

### Non-TCSPC-Based HT-FLIM

Time-gated approaches to FLIM involve recording the fluorescence decay kinetics in small sections of time (gates) and then combining those sections. This gives the same final result as TCSPC: a decay curve that represents the probability density function of photon arrival times used to calculate the fluorescence lifetime. These gates can be measured sequentially using one channel or simultaneously using multiple timing channels for higher acquisition throughputs. As a result, detector dead time is less of an issue than in TCSPC, but photon efficiency is lower since only part of the decay curve is being recorded at a time, though the multichannel techniques lessen this loss of photon efficiency. Analogous to integral approximation methods in calculus, having more time gates with smaller widths results in higher lifetime accuracy, particularly for fluorophores with short lifetimes or multiexponential decay, while having a longer overall detection time improves accuracy for longer fluorescence lifetimes [46]. Adding more gates or increasing the detection window size leads to longer acquisition times and lower throughput; however, analysis algorithms [21, 47] and even deep learning [22] have been implemented to maintain accuracy while collecting less images or gates. Similar detectors used for TCSPC can be used in time-gated FLIM, including modified SPAD arrays with gating electronics.

Select methods that do not fall under the main umbrellas of TCSPC or time gating are direct waveform recording (DWR) and frequency-domain FLIM (fd-FLIM). Similar to TCSPC and time gating, a pulsed laser source and common detectors, like PMTs, are used, but instead of the typical timing electronics following the detector, a high-speed digitizer records the entire analog waveform coming out of the detector, largely avoiding the issue of dead time inherent in photon counting. One recent application of DWR can be found in a fluorescence lifetime microplate reader using a high-intensity laser to allow for a fast and accurate lifetime recording and FRET recording, covering a 384-well-plate in 3 min in the early designs and, more recently, a 1,536-well-plate in the same amount of time [6, 29, 30, 32]. Generally being a simpler option to acquire fluorescence lifetime images at high frame rates, fd-FLIM remains viable for those who require speed over the high accuracy or sensitivity. Some recent implementations of fd-FLIM include an automated wide-field system built from commercial parts for FRET analysis of samples in 96-well-plates or microscope slides [28] and the use of a modulated laser in light-sheet microscopy with a CMOS camera to perform real-time, 3D fluorescence lifetime imaging [48].

### Multiphoton Excitation

**FIGURE 4 |:**
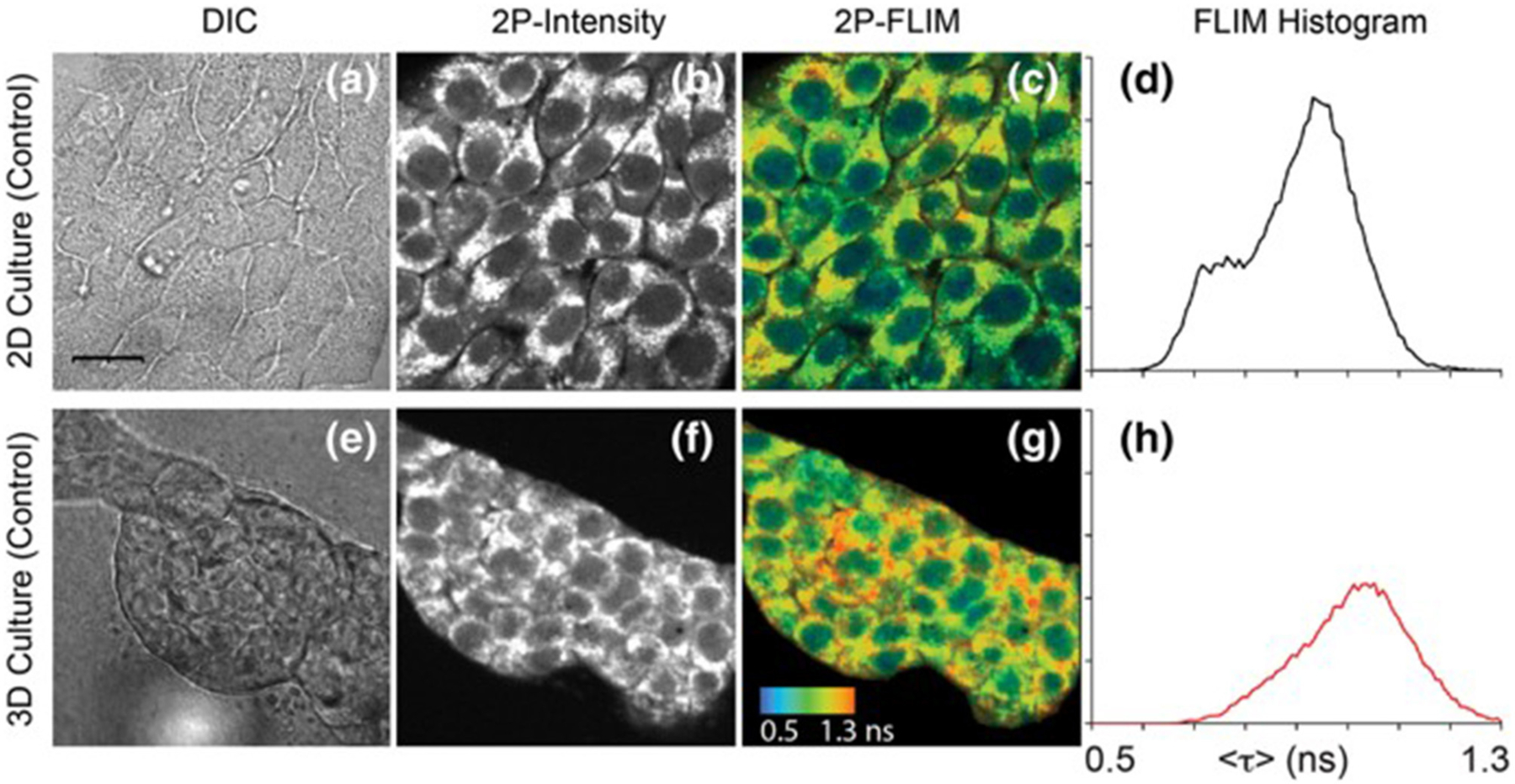
Images of NAD(P)H in 4T1 cells taken from a 2D monolayer culture and 3D matrix culture showing differential interference contrast microscopy **(a,e)**, two-photon fluorescence intensity **(b,f)**, and two-photon FLIM **(c,g)** along with histograms of the fluorescence lifetimes from the FLIM images **(d,h)**. These images highlight the usefulness of two-photon excitation to image live cells *in vivo* as there were differences in cell metabolism, as indicated by NAD(P)H fluorescence, between cells cultured in a typical lab environment (2D monolayer) and those cultured in a more realistic environment (3D culture). Adapted with permission from [20] © 2018 International Society for Advancement of Cytometry.

Another notable facet of FLIM technologies is the capability of multiphoton excitation. Traditional FLIM systems use one-photon excitation, where the absorption event of one photon provides the energy required to raise an electron to an excited state. However, as first theorized in 1929 and later proven in 1961, it is possible for two (or more) lower-energy, longer-wavelength photons to simultaneously interact with an electron to result in excitation [49]. This results in a number of benefits, such as less damage and deeper penetration in live samples, given the longer excitation wavelength, greater spectral separation between the excitation light and emission light, and the inherent ability to perform optical sectioning due to the non-linear relationship between excitation light intensity and multiphoton absorption [50]. Since much greater light intensity is required to make up for the lower probability of more than one photon simultaneously being absorbed, multiphoton excitation typically employs the use of highly focused, femtosecond-pulsed lasers. This allows for moments of very high intensity and low overall laser power felt by the sample and, consequently, is already an ideal excitation setup to perform FLIM and can utilize the lifetime detection methods discussed previously. Due to the nature of the advantages fundamental to multiphoton excitation, applications often involve *in vivo* imaging (see Figure 4), viewing dynamic biological processes over time, or imaging fluorophores with short-wavelength absorption within delicate live samples [13, 17, 19, 20, 26, 51].

## TIME-RESOLVED FLOW CYTOMETRY

**FIGURE 5 |:**
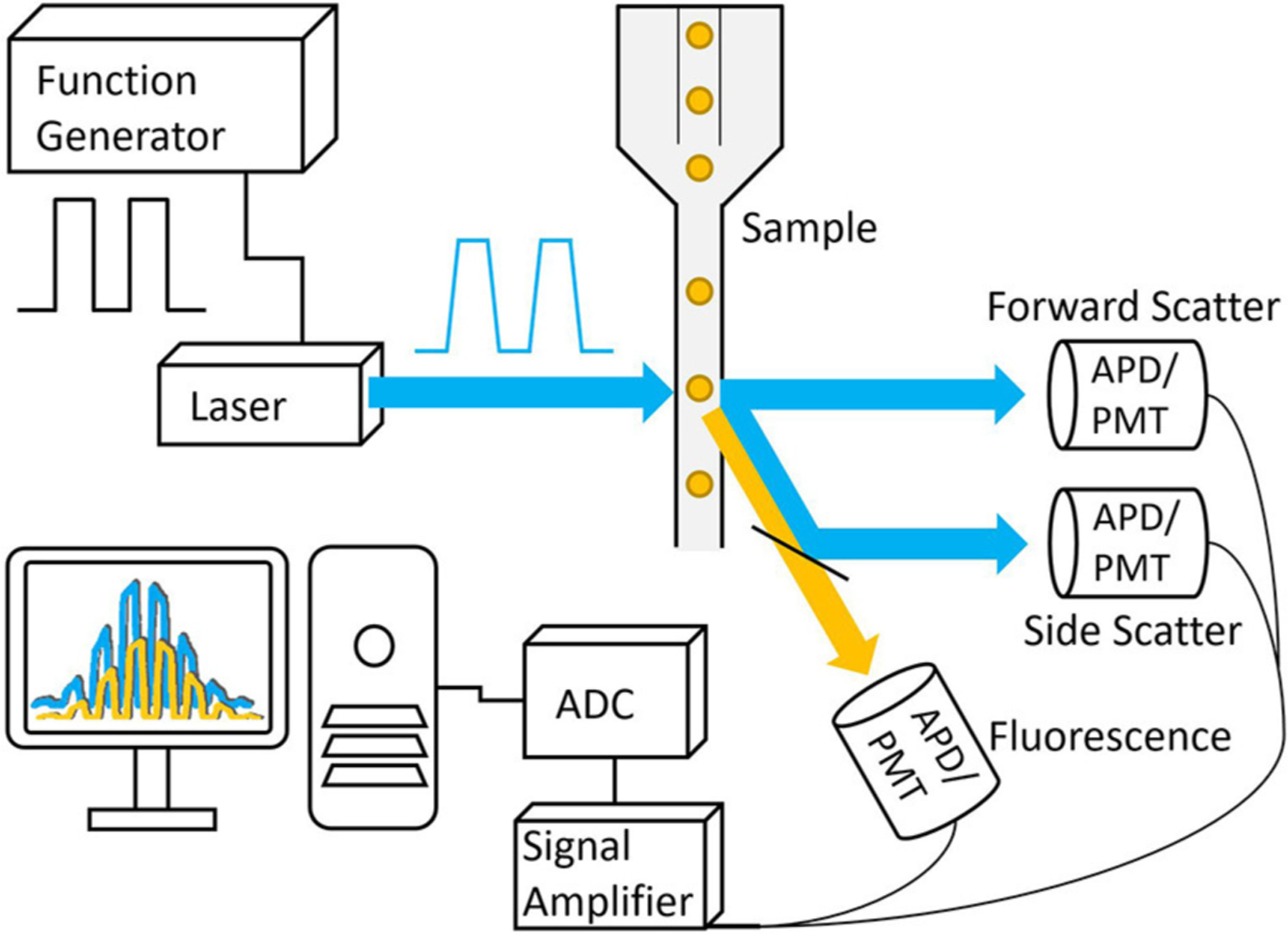
Basic schematic of a frequency-domain TRFC system. A function generator is used to modulate the laser that is then focused onto a small interrogation point in the flow path of the sample. The scattered excitation light and fluorescence emission light are directed toward the detectors, producing analog signals that are amplified and digitized by the analog-to-digital converter.

Time-resolved flow cytometry remains a niche field when compared to FLIM, mainly owing to the high-throughput nature of flow cytometry methods. TRFC was first demonstrated in the 1990s in the form of “phase-sensitive flow cytometry” (PSFC) [52, 53]. PSFC used analog phase-sensitive detection electronics, in which the excitation source and photodetectors were homodyned to resolve phase shifts between the modulated excitation source and the fluorescence emission (see Figure 5). The amplitude-demodulated fluorescence signal was phase-shifted to π/2+φ_1_ with respect to the reference signal to directly calculate the fluorescence lifetime value. This concept was demonstrated using Chinese hamster ovary (CHO) cells that were exogenously labeled with fluorescein isothiocyanate (FITC) and propidium iodide (PI). After binding, a mean lifetime of 3.5 ns was reported for FITC, whereas the mean lifetime of bound PI was measured to be at 15.0 ns. Digitalization of time-resolved waveforms shortly followed [54], allowing for more rapid analysis including the introduction of dual-frequency analysis [55] for improved single-cell analysis and the first chromophore quenching study in TRFC [56]. DNA content was analyzed in viable cells with exogenous DNA-binding probes [57], bringing about cell-cycle analysis using TRFC, and it progressed to the analysis of fixed cells to quantify and discriminate DNA and RNA content [58, 59]. TRFC emerged from NFCR during the late 2000s, and the niche technology began to mature and became a more versatile technology that focused on overcoming limitations found in conventional flow cytometry [60].

### Frequency-Domain TRFC

**FIGURE 6 |:**
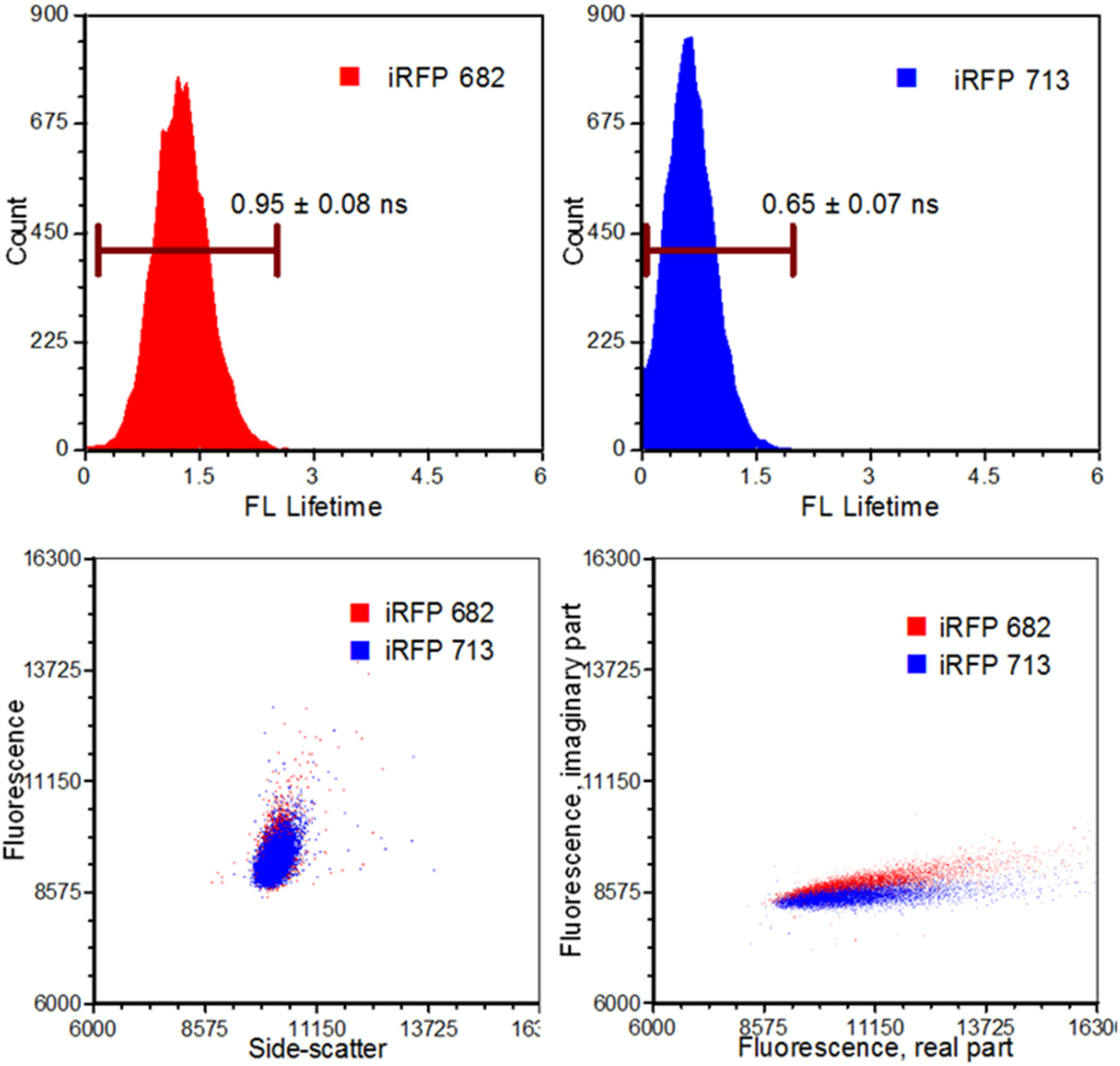
Results from an infrared fluorescent protein study (iRFP). Left, top: An iRFP with an emission maximum of 682 nm expressed a fluorescence lifetime value of 0.95 ± 0.08 ns. Right, top: An iRFP with an emission maximum of 713 nm expressed a fluorescence lifetime value of 0.65 ± 0.07 ns. Bottom, left: Dot-plot illustrating fluorescence vs. side-scatter for both the iRFP populations. Conventional fluorescence-activated cell sorting applies population gating to sort and enrich populations of interest. Significant overlap would inhibit users from achieving high sort yields. Authors from Yang et al. [62] applied time-resolved sorting to achieve maximum sort yields. Time-resolved data is processed by a fast Fourier transform and is transposed to frequency spectrums. Direct current and modulating frequencies are identified to calculate the phase value on a per-event basis. The subtle differences with respect to phase values between the two iRFPs illustrate two distinctive populations that permit sorting. Figure adapted with permission from The Optical Society ® [62].

The advancements in electro-optical technologies and instruments have facilitated a currently widespread use of frequency-domain flow cytometry (fd-FCM) systems [39]. Some examples of these advancements include the ability to directly modulate a solid-state laser at radiofrequency, modular high-speed transimpedance amplifiers, high-speed analog-to-digital conversion with field programmable gating array (FPGA) capabilities, and the overall size reduction of processing hardware [40]. The main advancement that has benefited from TRFC is the data-acquisition systems with digital sampling rates in excess of 100 mega samples per second (MSPS). Using these systems enabled researchers to perform TRFC for lifetime analysis as well as sorting with modified commercial systems [4, 7, 39]. For example, sorting based on the fluorescence lifetime was made possible using digital signal processing (sort purities >90%) as well as the development of pseudophasor analyses [4, 7, 61]. The sorting feasibility study which was performed involved separating yeast cells that expressed fluorescence proteins (XFPs) having co-spectral emission [7]. That is, different XFPs with similar emission spectra were measured and separated based on their fluorescence lifetime values. The observed difference is illustrated in the pseudophasor graphs given in Figure 6.

**FIGURE 7 |:**
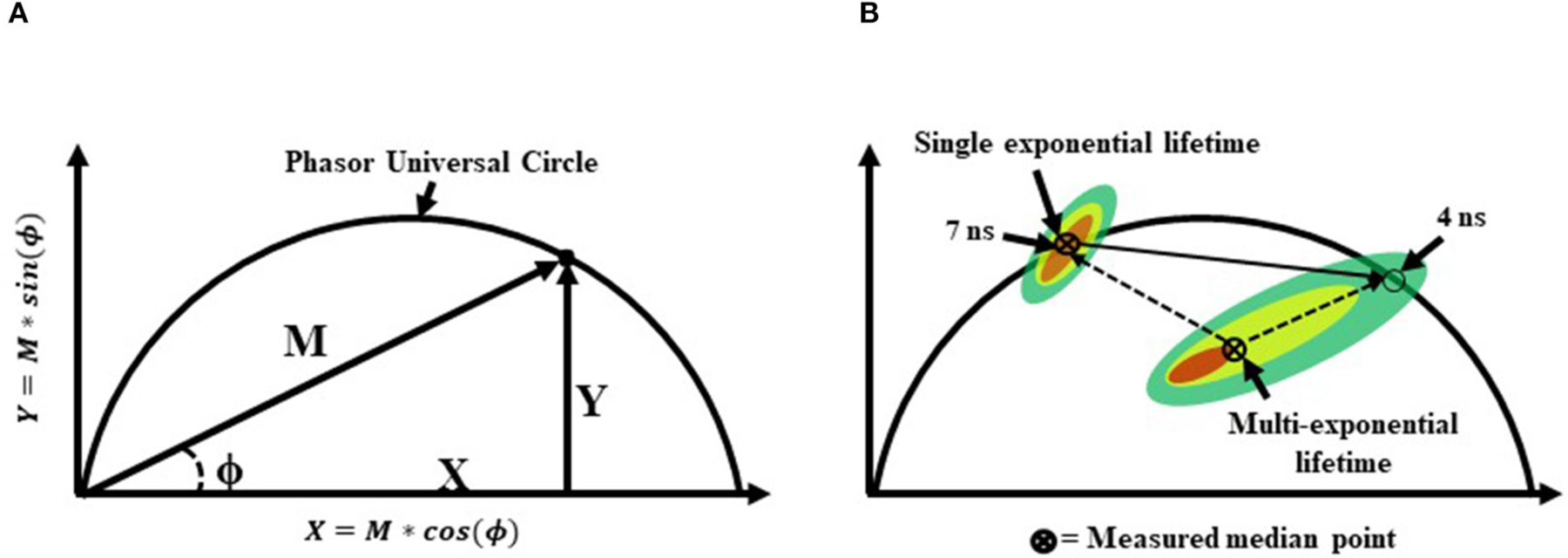
**(A)** A graphical representation of a phasor graph. Raw data is transposed to the frequency domain by applying a discrete Fourier transform. Data are visualized in a frequency spectrum where the phase (ϕ) and modulation (M) factors are extrapolated to construct the phasor graphs. Calculated events that fall directly on the phasor universal circle represent a single exponential lifetime, whereas calculated events falling within the phasor universal circle represent multiple exponential lifetimes. Alternatively, it is possible to gain insight into the weighted mean lifetime by calculating fractional contributions of different lifetime components using lifetime component vectors as represented in part **(B)**.

**FIGURE 8 |:**
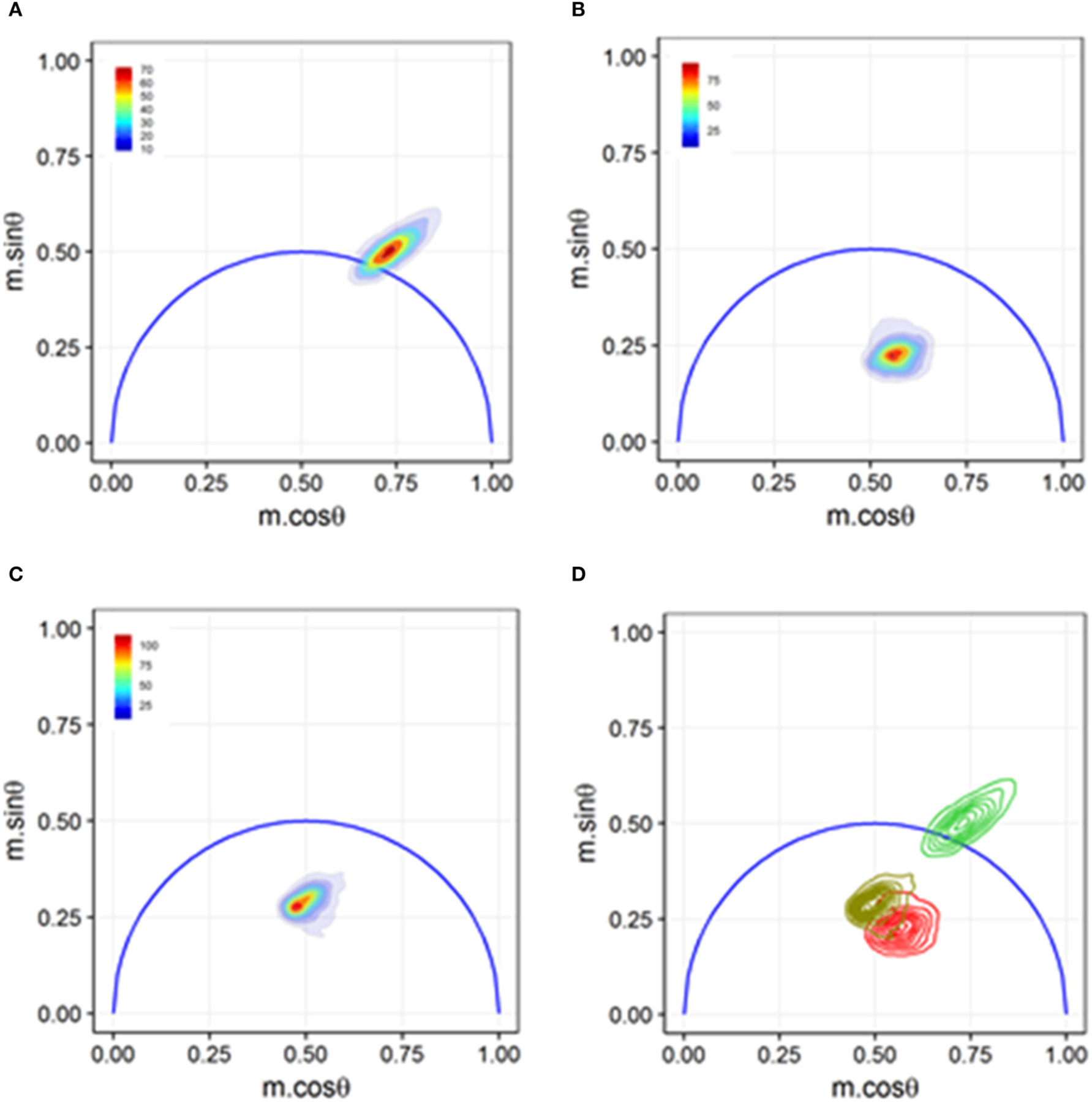
Phasor graph analysis for a Förster resonance energy transfer (FRET) study that resolved integrin conformational changes in the presence of artificial stimuli. **(A)** Transfected U937 ΔST cells were labeled with conjugated donor probe Leucine-Aspartic Acid-Valine binding residue conjugated to fluorescein isothiocyanate (LDV-FITC). The LDV residue has an affinity for the binding site on the inactive α4 integrin. **(B)** Acceptor probe PKH-26 (red, lipophilic fluorescent cell membrane dye), which binds to the cell membrane, was introduced to the cell suspension. Immediate quenching occurs because of the proximity of the donor probe to the acceptor probe. The population transits to the center of the phasor graph, indicating the presence of multiple fluorescence lifetimes as some cells are undergoing FRET and some are unaffected. **(C)** Artificial stimuli formyl Methionine-Leucine-Phenylalanine-Phenylalanine (fMLFF) elicits activation of α4 integrins. Integrins extend to their activated state, effectively removing the donor probe out of the proximity of the acceptor probe. Significant heterogeneity was present due to cell response or lack thereof to the artificial stimuli. **(D)** Contour plots illustrating the distribution and overlap of FRET and loss of FRET populations. Noticeable shifts were seen between the two populations, indicating a general response to the artificial stimuli. Figure adapted with permission from The Optical Society ® [31].

Sorting-based TRFC and non-sorting TRFC measurements both benefit from the use of phasor analysis (Figure 7). Phasor graphing is quite useful to TRFC because a phasor plot is comparable to a cytometry scatter plot. Scatter plots are useful for setting sort gates and for drawing conclusions about population distributions. Phasor plots were first introduced with FLIM data. With phasor analysis, the lifetime data are coordinate-transformed such that the magnitude of the demodulation value is represented as a magnitude (point) in the phasor space, and the phase-shift value is represented by the angle of the vector relative to the horizontal axis of the phasor graph. With TRFC, each point on the phasor graph is representative of the signal from a single cell; thus, for each cell, the intensity-weighted average of all present lifetime components [8] is considered. If the weighted value falls at the outer semi-circle, the signal obtained is a single fluorescence lifetime, not a combination of more than one. When the weighted phasor value for a given cell is observed to be inside the semi-circle, it can be inferred that multiple decay kinetics are present. Thus, the value represents the presence of multiple fluorescence lifetimes [63]. The robustness of phasor graphing was demonstrated in a TRFC FRET integrin study where surface integrins varied in their conformational states in the absence and presence of artificial stimuli. The study found the existence of multiple photophysical states of an antigen-conjugated chromophore that were not easily resolved with TRFC alone (see Figure 8) [31]. Phasor analyses can generally improve TRFC data collection and provide a way to statistically map lifetime changes, which is important for many biological applications.

The many single-cell analysis and sorting applications that have benefited from the use of TRFC include both digital frequency-domain and time-domain (described later) approaches. Some examples of frequency-domain TRFC include the measurement of fluorescence protein translocation within cells. TRFC measurements of protein movement were studied in the context of autophagy [25], in which the lifetime of enhanced green fluorescence protein (EGFP) when bound to the LC3 protein was affected depending on how the fusion protein was sequestered inside the cell. Thus, the lifetime was an indirect measure of the punctae localization of EGFP-LC3 into autophagosomes. In other examples, TRFC was used to study enzyme activity during apoptosis with the aid of tunable-FRET bioprobes [27]. The goal of the specialty bioprobes was to enable quantitative analysis of enzyme activity for high-content screening toward the discovery of potent anti-cancer agents. This work was later extended by the inclusion of TRFC phasor analyses, which provided statistical “fingerprints” of the loss of FRET, which was correlated to caspase enzyme activity at the onset of apoptosis [34]. In other fd-FCM studies, apoptosis-dependent phagocytosis of bacteria-inoculated cells was examined to determine if EGFP lifetimes could be used as a pH indicator, since pH changes are often expected in the phagosome microenvironment during phagocytosis of bacteria cells by macrophages [9]. A decrease in the EGFP fluorescence lifetime by ~1 ns was measured with fd-FCM as EGFP-labeled bacteria were taken in by the macrophage cells. Finally, in more recent studies, lifetime changes in the metabolic cofactor, NAD(P)H, have been measurable with TRFC. These metabolic mapping studies contribute to a growing body of FLIM research on the bound and free states of NAD(P)H, which influences the decay kinetics of this endogenous fluorophore. When measured with a cytometry system, the NAD(P)H fluorescence lifetime can be screened for large numbers of cells, thereby revealing the heterogeneity in the metabolic state from cell to cell [18]. The lifetime measurement of NAD(P)H as well as flavin adenine dinucleotide is common in FLIM and is now being studied using fd-FCM.

### Time-Domain TRFC

Time-resolved flow cytometry with time-domain systems has also been designed and optimized for a variety of biological applications. Time-domain flow cytometry (td-FCM) systems excite cells with consecutive excitation pulses, and the fluorescence decay is measured over a given time resolution. These cytometers perform photon counting with optoelectronics that capture photons and bin them with respect to their arrival time [64]. As discussed previously, in time-domain FLIM, the systems that acquire time decay kinetics can be slower than their frequency domain counterparts due to dead times between detection windows. In td-FCM, pulse back-up occurs, resulting in the counting of losses as the event rate increases. However, in the time domain, multiple lifetimes can be readily calculated cell by cell. In a recent example, td-FCM was demonstrated with PMT detectors, TCSPC control modules, and microfluidic-based flow control. This work, by Nedbal et al., captured photons in 20 μs time bins and fit data with exponential decay models. The throughput of this system reached approximately 3,000 events min^−1^; in contrast, fd-FCM systems count at rates up to 60,000 s^−1^. However, in 2016, advancements were made with respect to CMOS sensors, providing higher-resolution timing through embedded FPGAs and reducing data transfer rates and CPU power needed to process raw data. The implementation of a CMOS SPAD array in conjunction with a series of eight time-interleaved TDCs greatly reduced signal pile-up, allowing for a maximum sample rate of 60,000 s^−1^ with very low error rates [65].

Other unique hybrid td-FCM systems have been developed in order to enhance the ability of a cytometer to capture multiple-decay kinetic signals while retaining a high event rate. One example of this was a td-FCM system in which the excitation source was “pulsed” over the moving cell multiple times with the aid of acousto-optic deflection [5]. The laser was “rastered” across the cell several times in the span of 25 ns, enabling the ability to acquire multiple single exponential decay and dual exponential decay waveforms per cell in a single spectral channel. To resolve the decay kinetics of the excited fluorophores, a post-processing deconvolution analysis was applied to the scatter and fluorescence waveforms. If a single exponential decay is assumed to be present, a convolution of the Gaussian function gives the exponential curve of the former Gaussian curve, allowing the calculation of the fluorescence lifetime value. If multiple decays are present, re-convolution is performed using the Fourier convolution theorem. A Gaussian function is first convoluted with the proper multi-exponential decay model and then deconvoluted to separate instrument response from species that contribute to the measured fluorescence lifetime. In similar examples, digital signal processing (DSP) approaches were used to calculate the fluorescence lifetime in the time domain by estimating the time delay between a fluorescence and scattered light signal [61, 66]. Light pulse signals collected from conventional cytometry (forward scattered light waveforms and fluorescence waveforms) were compared offline [61] with a variety of interpolation or fitting algorithms to prove that this value correlates to the average fluorescence lifetime [61]. Other advanced processing approaches were demonstrated by using a modified chirp Z-transform (MCZT) to acquire high-resolution spectral data. This was followed by a fine-interpolated correlation peak (FICP) algorithm applied and integrated with a time-domain cross-correlation function to permit the calculation of time delay between the forward scattered light and fluorescence signal [66].

Sample isolation and enrichment through fluorescence-activated cell sorting (FACS) is also possible with td-FCM using a technique known as fluorescence-activated droplet sorting (FADS). A recent study employed TCSPC [67] for oil droplet-based microfluidic sorting and sample encapsulation. The sample was measured based on the emitted spectra and fluorescence lifetime value. Sort decisions were made using multiparametric information. Fluorescence intensity and lifetime measurements within an acceptable value triggered the FPGA of the data acquisition system to send a signal to a function generator that activated dielectrophoresis electrodes to isolate the cells that fall within the targeted threshold. Sort purities achieved for mScarlet and mCherry RFPs at a sort rate of 2,500 events min^−1^ were 80 ± 1% and 97 ± 1%, respectively. Furthermore, Hung et al. demonstrated an approach that would permit the encapsulation of multiple cells per droplet, permitting the system to achieve sorting times for RFP libraries (~108 events) within 3 h, which is three times faster than state-of-the-art cell sorters.

Aside from fluorescence-based measurements, it is possible to acquire longer luminescence lifetime values with slightly altered electronic and td-FCM instrumentation setup. As a recent example of this, autofluorescence crosstalk was resolved using Europium-doped polystyrene beads and THP-1 cells. A td-FCM system was designed to measure the long lanthanide lifetimes of Eu (range of μs or ms). The luminescence decay was long enough to not interfere with shorter fluorescence signals [68]. The long-lifetime td-FCM instrument measured the focused cells acoustically, and the throughput was lower compared to conventional cytometry. Similarly, Kage et al. [69] developed a td-FCM system to measure the lifetime values of four types of quantum-dot-doped polymer beads. In this study, it was demonstrated that longer lifetimes of the quantum-dot-doped beads can be discriminated from shorter lifetimes found in dye-based beads by measuring the respective decay by separate tempo-spectral channels.

## DISCUSSION (FLIM AND TRFC CROSSOVER AND FUTURE)

**FIGURE 9 |:**
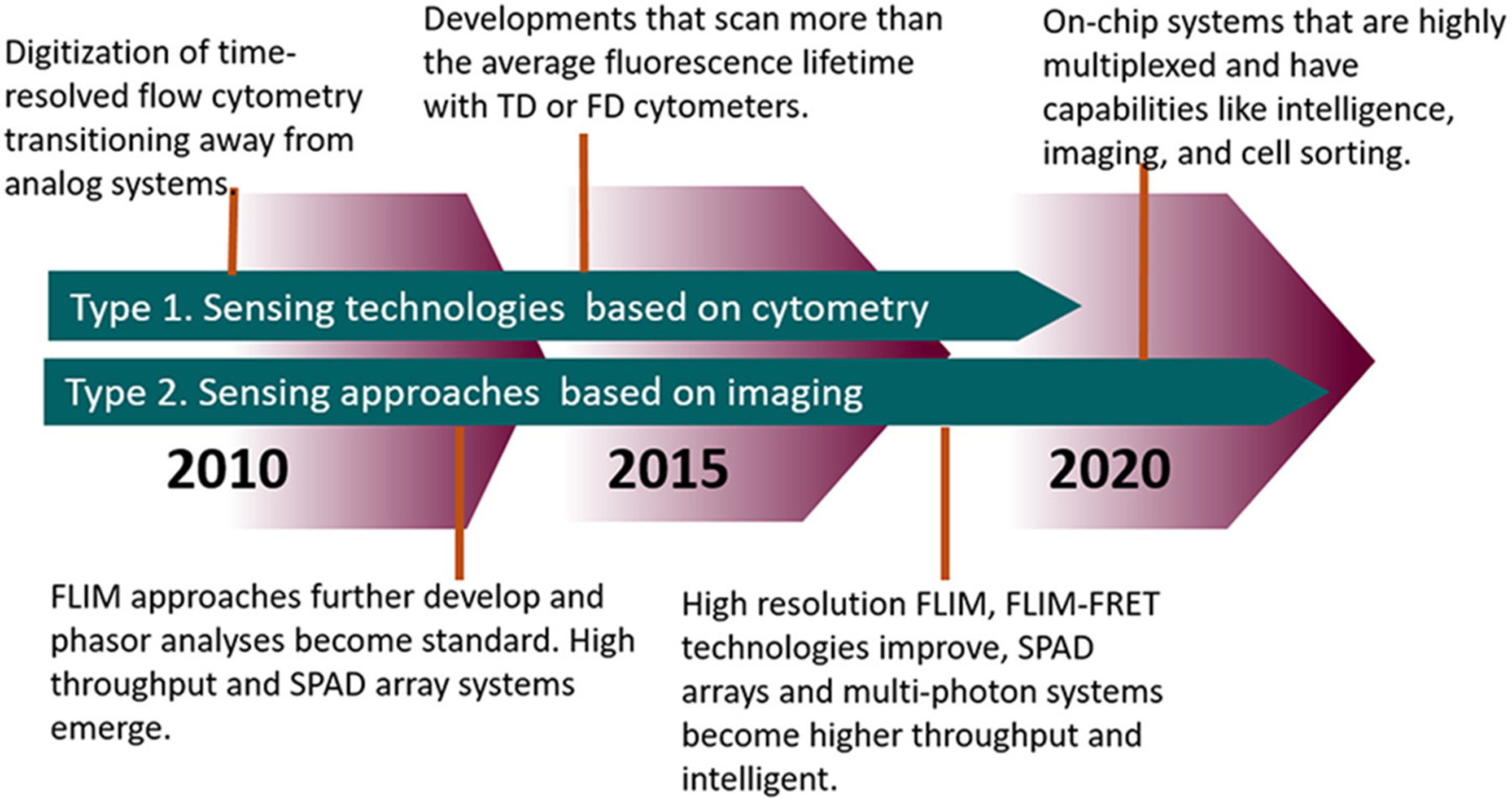
Timeline highlighting the advances in FLIM and TRFC from the past decade and moving forward.

The measurement of fluorescence lifetime is becoming an increasingly useful and necessary tool for studying biological characteristics and phenomena from the tissue level down to the protein level. Tables 1, 2 highlights modern innovations in fluorescence lifetime instrumentation and applications, which are also depicted by the flow diagram in Figure 9. Many of the limitations in using fluorescence lifetime are related to throughput, particularly in the realm of detection hardware and computational processing power. This review discussed the evolution of fluorescence lifetime techniques in the fields of FLIM and TRFC, which are technologies that are raising the bar for high throughput and high content analysis. Currently, there are still pitfalls (i.e., resolution, accuracy) to increasing the speed of lifetime acquisition. Nonetheless, advances in technology and software hint at a future in which the fluorescence lifetime will be precisely measured at a reasonable throughput without much compromise.

The future of high-throughput FLIM and cytometry is indeed foreshadowed by novel machinations of deep learning, imaging, multiplexing, and intelligent hardware. Use cases in which lifetime imaging cytometry is possible are emerging (e.g., FLIM systems that capture multiple fluorescence parameters simultaneously at a high throughput [10]). Deep learning is also trending as it can increase the speed of lifetime analysis by reducing the number of images acquired without compromising accuracy or resolution [22, 24]. Hybridization of instruments, such as combining multiphoton wavelength-swept excitation, diffraction grating, and galvanometric beam scanners to perform rapid multiplexed imaging, is also advancing this field [13]. Many opportunities are possible with these technologies, as well as SPAD arrays and other high-speed, high-resolution time-domain lifetime detection methods [2, 65].

The future of high-throughput fluorescence lifetime will involve huge advancements in instrument and data analysis. These advancements will likely be built upon existing, established methods as well as novel innovations in instrument speed and resolution. The physical tools that enable high-throughput lifetime analyses will perhaps uncover traits of cells, cellular processes, and other biochemical interactions that otherwise are not currently detectable. The eventual dissemination of such tools will certainly influence bioscience and will lead to eventual clinical translation for a true biomedical impact.

## Figures and Tables

**TABLE 1 |:** Common fluorescence lifetime sensing techniques.

Technique	Theory/Principle	Detection method	Advantages	Limitations
TCSPC (Time domain)	Timing of fluorescence photons is measured against a pulsed excitation source and organized into a histogram representing the fluorescence decay curve.	Timing electronics paired with detectors (PMTs, hybrid PMTs, APDs), or silicon-based detectors with built-in timing circuitry (SPADs with TDCs).	Very high accuracy and temporal resolution	Photon pile-up/detector dead time. Lower throughput.
Time gating (Time domain)	Similar to TCSPC, but only small sections of the fluorescence decay curve are measured at a given time or across a given detection channel.		Limits or eliminates issues of pile-up and/or dead time for faster acquisition.	Typically less sensitive and/or accurate compared to TCSPC.
Direct waveform recording	Entire fluorescence waveform is captured and used to extract the fluorescence lifetime.	Fast detectors paird with high-speed digitizers to convert the analog signal real-time.		
Frequency domain	Decay time results in a phase shift between fluorescence and excitation source that is directly proportional fluorescence lifetime and can be calculated using Fourier transforms.	Function generators required to modulate excitation source. Standard fluorescence detectors and high speed data acquisition systems.	Continuous detection with very high throughput.	Accuracy, resolution, or both are more dependent on fluorophore brightness.

**TABLE 2 |:** Fluorescence lifetime measuring technology and methods in the past decade.

References	Technology and methods	Brief summary
Yuan et al. [70]	Fluorescence spectrometer, AOTF, optical biopsy	AOTF and collection of first-order diffraction beams. Acquisition of 200 nm time-resolved spectra in 4 s.
Houston et al. [39]	Frequency domain TRFC, lifetime-based sorting, ORCAS	Open reconfigurable cytometric acquisition system (ORCAS) adds capability to perform lifetime analysis on any cytometer with a laser that can be modulated.
Tyndall et al. [42]	TCSPC, SPAD Array, integrated silicon photomultiplier (SiPM)	Parallelization of TCSPC to overcome photon pile-up. CMOS process used to make a SiPM with SPAD array, TDCs, and lifetime estimation on-chip.
Li et al. [5]	fd-TRFC, fluorescence lifetime excitation cytometry by kinetic dithering (FLECKD)	Rapid scanning of laser across sample passing through flow cytometer. Able to discriminate multiple fluorescence lifetimes simultaneously.
Petersen et al. [6]	High throughput fluorescence lifetime plate reader. Direct waveform recording (DWR)	Waveforms are direclty digitized for lifetime calculation. Fluorescence lifetime plate reader can image 384-well microplate in 3 minutes with better than 1 % accuracy.
Nedbal et al. [2]	TCSPC, microfluidic FLIM, Burst-Integrated Fluorescence Lifetime (BIFL)	Epifluorescent microscope with associated BIFL software used to determine intensity of fluorescence, photon rate, lifetime, and burst duration for each cell.
Poland et al. [26]	Multifocal multiphoton FLIM (MM-FLIM), SPAD array, TCSPC, FRET	Parellelized MM-FLIM in both excitation and detection. Technique showed increased speed in comparison to confocal FLIM and widefield FLIM.
Rocca et al. [65]	TCSPC, CMOS SPAD array, SiPM, BIFL, Field Programmable gate arrays (FPGA)	A single-chip is equiped with SiPM capable of BIFL using TCSPC for detection and real-time sorting with FPGA using CMM for lifetime calcuation.
Lee et al. [15]	Phasor-FLIM based single cell screening	Single-cell traps within a microfluidic device allow for differentiation of cells based on metabolic differences in NAD(P)H without any labeling using phasor-FLIM
Mikami et al. [71]	Frequency-division multiplexing (FDM) confocal microscope, imaging flow cytometry	Integration of a dual-frequeny comb that was spatially distributed along with QAM into FDM. 16,000 frames/s surpassed the fluorescence lifetime limit
Schaaf et al. [32]	Red-shifted FRET biosensors (OFP and MFP), high throughput screening/plate reader	The FRET pair developed increased efficiency, dynamic range, and signal-to-background of HTS. Can image 1536 well-plate in 3 minutes
Shen et al. [17]	Custom continuous-flow bioreactor, real time two-photon FLIM (2P-FLIM)	2P-FLIM was implemented to continuously monitor live cultures under shear stress, eliminating traditional interuptions of the bioreactor
Esposito and Venkitaraman [10]	Hyperdimensional imaging microscopy (HDIM)	Parallel detection of orthogonal fluorescence characteristics (lifetime, polarization, and spectra). Hyperdimensional traits detected with two multiwavelength TCSPC detectors.
Yao et al. [24]	A deep convolution neural network (CNN) called Net-FLICS (FLIM with compressed sensing)	Reconstruction of intensity and FLI maps using deep learning. Reconstruction times of <3 ms/sample, 4 orders of magnitude faster than previous methodologies
Hirmiz et al. [33]	FLIM-FRET combined technology with a highly-multiplexed confocal microscope	Microscope was coupled to an SPAD array for high resolution and rapid imaging of FLIM
Karpf et al. [13]	Spectro-Temporal Laser Imaging by Difracted Excitation (SLIDE), imaging flow cytometry	Non-linear microscope with kHz frame rate using a pulse-modulated, sweeping laser with inertia-free steering. Lifetime recording of 88×10^6^ pixels/s.
